# Is Primary HPV with Secondary p16/Ki67 Dual-Stain an Alternative HSIL-Risk Detection Strategy in Cervical Cancer Screening for Women under 30 Years?

**DOI:** 10.3390/diagnostics11112012

**Published:** 2021-10-29

**Authors:** Martyna Trzeszcz, Maciej Mazurec, Robert Jach, Karolina Mazurec, Zofia Jach, Izabela Kotkowska-Szeps, Magdalena Kania, Mariola Wantuchowicz, Anna Prokopyk, Piotr Barcikowski, Marcin Przybylski, Joanna Wach, Agnieszka Halon

**Affiliations:** 1 Corfamed Woman’s Health Center, Kluczborska 37, 50-322 Wroclaw, Poland; carlos.mazurec@icloud.com (K.M.); iza.corfamed@gmail.com (I.K.-S.); magda_kania@poczta.onet.pl (M.K.); mariolawantuchowicz@gmail.com (M.W.); ania.prokopyk@gmail.com (A.P.); barcikowski07@gmail.com (P.B.); asia03529@gmail.com (J.W.); 2 Division of Pathology and Clinical Cytology, University Hospital in Wroclaw, Borowska 213, 50-556 Wroclaw, Poland; 3 Division of Gynecologic Endocrinology, Jagiellonian University Medical College, Kopernika 23, 31-501 Krakow, Poland; jach@cm-uj.krakow.pl; 4 Superior Medical Center, Czyzynska 21/50, 31-571 Krakow, Poland; zofiajach@gmail.com; 5 Department of Obstetrics and Gynecology, District Public Hospital, Juraszow 7-19, 60-479 Poznan, Poland; nicramp@poczta.onet.pl; 6 Department of Clinical and Experimental Pathology, Division of Clinical Pathology, Wroclaw Medical University, Borowska 213, 50-556 Wroclaw, Poland; agnieszka.halon@umed.wroc.pl

**Keywords:** cervical cancer screening, high-risk HPV, HPV DNA, p16/Ki67 dual-stain, cancer biomarkers, CINtec PLUS, young women, triage

## Abstract

Recently, cervical cancer rates elevation has been noted in women aged 20–39 years in regions with a very high human development index (HDI). The onset of cancer elevation rates is observed in the age range of 25–29 years, which should necessitate effective precancer screening in younger age groups, including those <25 years. From 30.066 liquid-based screening tests results (*n* = 30.066), 3849 liquid-based cytology, 1321 high-risk human papillomavirus (HRHPV) and 316 p16/Ki67 performed in women <30 years were selected. Performance characteristics were calculated for three screening models: primary HRHPV with p16/Ki67 triage, primary cytology with reflex HPV and primary cytology alone. Primary HRHPV with p16/Ki67 triage was significantly more sensitive in high-grade squamous intraepithelial lesion quantified with cervical intraepithelial neoplasia grade 2 or worse [HSIL(CIN2+)] detection than cytology with reflex HRHPV and cytology alone (83.3% vs. 70.8%/45.8%) and had significantly higher diagnostic predictive values (PPV:29.4%/21.3%/22.9%; NPV:91.7%/82.9%/82.2%, respectively at CIN2+ threshold). The number of colposcopies per HSIL(CIN2+) detection indices was 3.4, 4.7 and 4.4, respectively. Primary HPV testing in women <30 years with p16/Ki67 triage of HPV-positive cases might be an effective cervical cancer screening strategy for HSIL(CIN2+) detection with superior diagnostic performance when compared with primary cytology-based models. Women <25 years might also benefit from an introduction to a more sensitive screening approach.

## 1. Introduction

The incidence and mortality of cervical cancer vary globally depending on the geographic region and the level of the HDI [1]. In countries with a very high HDI, there has been an increase in cervical cancer incidence in recent years, and this applies to women in the 20–39 age group [2], which is a worrying phenomenon. The onset of cancer rates growth is observed in the range of 25–29 years, which should draw the attention of prevention systems to women under 25 years of age and their effective screening for the precancers detection [2,3]. Vaccination against HPV, together with secondary prevention, are the important factors in reducing cervical intraepithelial lesions in young women [4,5].

Human papillomavirus DNA testing is the globally recommended primary screening strategy for cervical cancer prevention for women 25–65 years old in all resource settings [6] due to higher sensitivity, better reproducibility and less subjectivity compared with cytology [7,8]. The recommended age to start primary HPV or primary HPV-based screening according to the Australian and USA guidelines is 25 years [9,10,11]. Whereas the European guidelines for quality assurance in cervical cancer screening strongly recommend the introduction of the primary HPV model in women aged 30 or above, even above 35 [12]. Conversely, the newly released interim guidelines from Poland do not indicate a specific target age for beginning HPV-based screening while recommending the new triage option for HPV-positive women with p16/Ki67 dual-staining as one of the screening strategies [13]. In the vast majority of the available international guidelines, the recommended approach in patients aged 21 to 24 years is primary cytology-based screening, which results from high rates of transient HPV infections and associated abnormalities in this age range and may lead to overtreatment [14]. According to the first risk-based management guidelines for abnormal cervical cancer screening tests results and cancer precursors, young women aged 21–24 should be screened by cytology alone with reflex HPV testing in the cytologic diagnosis of atypical squamous cells of undetermined significance (ASC-US) [11].

Dual immunocytochemical staining in cervical cytology specimens with the simultaneous use of anti-proliferative p16 and Ki67 proliferative proteins is a morphologic-independent cell cycle dysregulation biomarker. A diagnostic value profile of p16/Ki67 testing is characterized by high sensitivity and high specificity for histologic high-grade intraepithelial lesions with evidenced effectiveness in HPV-positive women and/or with abnormal cytology results [15,16,17,18,19,20]. An approach with the introduction of p16/Ki67 dual-staining as a triage test for HPV-positive cases in primary HPV screening and for HPV-positive NILM cases in co-testing obtained approval from the Food and Drug Administration. An interpretation of the p16/Ki67 test should be performed by a qualified pathologist [21].

Data on the use of p16/Ki67 dual-stained cytology in women under 25 years are limited, especially when used as a secondary test in HPV-positive young women undergoing primary HPV screening. Due to increasing cervical cancer rates in women aged 25–29 years, we investigated in our study whether primary HPV screening with triage HPV-positive cases with the p16/Ki67 biomarker can be an alternative screening strategy to cytology-based screening in young women under age 30 years. For this purpose, we conducted a retrospective analysis of cytologic–virologic–immunocytochemical reporting rates with histologic correlation at this age range and an evaluation of the diagnostic value of three screening strategies for the HSIL (CIN2+) detection.

## 2. Materials and Methods

### 2.1. Study Population and Design

We conducted a retrospective analysis of cervical cancer liquid-based screening (LBS) tests results, including liquid-based cytology (LBC), 14 types of high-risk human papillomavirus testing (HRHPV14) and p16/Ki67 dual-stained cytology, undertaken at a private-based opportunistic cervical cancer screening at one of the largest Lower-Silesian private outpatient gynecological clinics (Corfamed Woman’s Health Center) from August 2015 to July 2020. There were three cervical cancer screening modalities used in the center over the period considered: cytology with reflex HRHPV14, co-testing (HRHPV14 simultaneously performed with LBC) and co-testing plus (co-testing simultaneously performed with p16/Ki67). The baseline analysis included a total of 30,066 screening tests results, including 20,605 LBC, 8331 HRHPV14 and 1130 p16/Ki67 tests sampled in women aged 15 to 92; mean age of 40.9. From the baseline group, all screening test results in women under age 30 were selected. Following the selection, 3849 LBC results, 1321 HRHPV14 and 316 p16/Ki67 tests results were obtained. The final study group consisted of 121 patients <30 years with available LBC, HRHPV14 and p16/Ki67 tests results who had colposcopy with biopsy and cytologic–virologic–immunocytochemical–histologic correlations could be assessed. Including all the LBS and histology results, in the final group, a retrospective analysis was conducted, and the diagnostic value was evaluated together with assessment numbers of colposcopies needed per detection of HSIL (CIN2+). This analysis comprised 3 screening models (the first screening round with unknown screening history), as follows: (1) model No 1 (M1)—primary HPV with reflex p16/Ki67 for all HPV-positive results; (2) model No 2 (M2)—primary cytology with reflex HRHPV, based on the risk-based management of ASCCP 2019 using a mobile application; (3) model No 3 (M3)—primary cytology alone without any reflex testing, based on the risk-based management of ASCCP 2019 using a mobile application. All analyzed data came from the center’s registry.

### 2.2. HRHPV Detection and Genotyping

HPV detection and genotyping were performed by the qualitative in vitro PCR Abbott RealTime High Risk HPV as recommended by the manufacturer, according to a report from the laboratory performing the test. This assay phenotypes 14 types of high-risk HPV DNA, including as follows (16, 18, 31, 33, 35, 39, 45, 51, 52, 56, 58, 59, 66 and 68) and specifically genotypes HPV 16 and HPV 18. If type 16 and/or 18 HPV is detected, the result was categorized as HPV16 or 18 or both positive. If one or more of the other 12 HRHPV14 types were detected, the result was categorized as N16/N18 positive.

### 2.3. Liquid-Based Cytology

Liquid-based SurePath slides were obtained using the automatic laboratory PrepStain Slide processor (Becton Dickinson, Franklin Lakes, NJ, USA) by a laboratory external to the center, and it was ensured that a laboratory procedure strictly adhered to the manufacturer’s protocol. All cytology samples were reported by a gynecological cytopathologist and classified according to the Bethesda 2014 guidelines. A cytopathologist was aware of the HRHPV14 status of the cervical samples. All residual cervical samples were stored for 6 months in the laboratory under the conditions specified by the manufacturer. This strategy enabled to perform additional tests also ordered later (including the p16/Ki67 triage) and with the patient’s comfort, who did not have to be called for another gynecological visit, and all cervical cancer diagnostics was performed from one cytological sample collection.

### 2.4. p16/Ki67 Biomarker

p16/Ki67 testing was performed in HRHPV14-positive cases and in women who underwent co-testing plus. Dual immunocytochemical staining of cervical samples was carried out using p16 and Ki67 proteins in CINtec PLUS detection kit (Roche, MTM AG laboratories, Monachium, Germany) processed in an automatic BenchMark XT laboratory system (Ventana Medical Systems, Inc., Oro Valley, AZ, USA), according to the manufacturer’s protocol, and with one control specimen present in each run. The p16/Ki67 testing was performed from the same LBC sample with residual material stored in a laboratory in the original SurePath vials (Becton Dickinson, Franklin Lakes, NJ, USA). An immuno-expression status was assessed by a gynecological cytopathologist specially trained and certified in p16/Ki67 review and classified as positive, negative, or unsatisfactory. A positive result of the p16/Ki67 test was the presence of at least one cell in the preparation meeting the following evaluation criteria: simultaneous red nuclear stain for Ki67 and brown cytoplasmatic stain for p16 in the same epithelial cell. For a morphological evaluation of the cell groups, the positive diagnosis was determined by a strong diffuse p16 stain within the group and the presence of at least one cell with a Ki67 stain in the nucleus and a p16 stain in the cytoplasm at the edge of the assessed group and/or sheet of cells. The negative p16/Ki67 test result was defined when no staining or single staining of p16 or Ki67 was present in the epithelial cell. An unsatisfactory p16/Ki67 test result was determined by features included in the manufacturer’s kit and/or by cellularity criteria, which are consistent with the criteria for a liquid-based cytological preparation.

### 2.5. Colposcopic Biopsy and Histology

Women with positive p16/Ki67 test result, ASC-US or LSIL HRHP14 positive, with cytologic ASC-H or HSIL regardless of HRHPV14 status, or with ASC-H or HSIL without HRHPV14 (in cytology alone screening model) were referred for a colposcopy. Indications for the colposcopy were according to Polish guidelines with the extension of the 2012 ASCCP guidelines and 2015 ASCCP interim guidelines. The colposcopy protocol included endocervical sampling as a minimum sampling approach and additional direct or random biopsies if required. Histologic diagnoses of cervical biopsies and endocervical specimens (obtained using curettage with brushing) were reviewed by a gynecological pathologist, according to the LAST 2012/WHO 2014 terminology [22,23]. Colposcopies performed outside the center, histologic reports evaluated by other than the center’s pathologist due to different colposcopy protocols, different histologic nomenclature and/or lack of recommended p16 immunohistochemistry used in histologic reports were not included in the study.

### 2.6. Statistical Analysis

Statistical analysis was conducted using licensed PQStat Software. A diagnostic value of tests, measured with sensitivity, specificity, positive (PPV) and negative (NPV) predictive values, and positive (PLR) and negative (NLR) likelihood ratios, was calculated according to standard definitions. Differences in diagnostic value between the analyzed screening models were evaluated, and the colposcopy number needed for each model was also compared. A *p* < 0.05 was considered significant.

## 3. Results

In the analyzed period, from August 2015 to July 2020, a total of 5.486 LBS tests were performed in patients aged 15–29 years, with a mean age of 25.3 years. A total of 3.849 LBC results were analyzed, with 1.321 HRHPV14 and 316 p16/Ki67 tests results. The following reporting rates were obtained for cytological diagnoses compared to the group of women over 30 years of age, NILM, ASC-US, LSIL and ASC-H+ (% <30 years of age/% ≥30 years of age) 91.3/94.7, 3.9/3.2, 4.2/1.5 and 0.6/0.57, respectively. The biggest difference in the baseline group <30 years of age was for diagnosis of LSIL, which in women <30 years of age was almost three times more frequent than in the 30+ group. In the final study group of 121 women, detected HSIL (CIN2+) cases were 19.83% of all histologic diagnoses. No screening in this group of women would lead to remain these HSIL (CIN2+) lesions undetected, with all the potential consequences. Table 1 and Table 2 present the complete results in this regard stratified by age. A total of 24 HSIL (CIN2+) cases (6 cases in patients <25 years and 18 cases in the 25–29 age group) were detected in the study group. No case of HSIL (CIN2+) HRHPV14-negative was found in the study group.

Table 3 and Table 4 present the complete data for M1. When using screening model 1, four cases of HSIL (CIN2+) HRHPV14-positive and p16/Ki67-negative would not be detected, and the number of colposcopies needed would be 68.

Table 5, Table 6 and Table 7 present the complete data for M2 and M3. When using screening models 2 and 3 would not have detected 7 and 13 HSIL (CIN2+) cases, respectively, and the number of colposcopies needed would be 80 and 48. When using M3, all cases of HSIL (CIN2+) in women aged <25 years with preceding ASC US and LSIL in cytology would not be detected in the first screening round (surveillance with control cytology in 1 year is recommended for these cases)—three cases (12.5% of all) in the analyzed group. When using M2 and M3 screening models, all HSIL (CIN2+) cases with preceding NILM diagnosis would be missed—two cases in the age group <25 years and two cases in the age group 25–29 years in the study. Whereas using M1 in women <25 years with NILM, ASC-US and LSIL cytologic results, it would detect 80% of HSIL (CIN2+) cases in this group—one case was HRHPV14-positive and p16/Ki67-negative. These detected HSIL (CIN2+) represented 16.7% of all HSIL (CIN2+) cases in our study. M1 used in the group of women with preceding NILM cytology diagnosis would detect two HSIL (CIN2+) cases in age group <25 years (included above) and two cases in women aged 25–29 years, all these patients were HRHPV14-positive and p16/Ki67-positive. In total, screening model 1 used for NILM, ASC-US and LSIL results in women <25 years and NILM in women aged 25–29 years would detect six HSIL (CIN2+), which accounted for 25% of all cases in the group <30.

The highest level of the diagnostic value, including sensitivity, PPV, NPV, PLR and NLR, was in screening model 1 of 83.3, 29.4, 91.7, 1.60 and 0.35, respectively. Only in specificity, the highest value of 61.9% was in screening model 3, but it should be remembered that this would be at the expense of not detecting 13 cases of HSIL (CIN2+). In M3, the lowest absolute number of colposcopies needed to perform would also be obtained, but the detection index (number of colposcopies needed per HSIL (CIN2+) detection) is significantly higher in M3 than in M1. The results of the diagnostic value for the analyzed models, a number of HSIL (CIN2+) missing cases and a number of colposcopies performed in each model along with detection of HSIL (CIN2+) vs. colposcopies needed are presented in Table 8, Table 9, Table 10, Table 11, and Table 12 respectively.

## 4. Discussion

In this study, we compared the diagnostic value of primary HPV screening with p16/Ki67 triage and cytology-based screening strategies (cytology with reflex HPV testing or cytology alone; in both, the management was based on ASCCP 2019 guidelines) in women under 30 years of age. The sensitivity of screening model 1 (primary HPV with p16/Ki67 triage) was significantly higher than the sensitivity of cytology with reflex HPV and cytology alone screening models for detection histologic HSIL (CIN2+) in this age range (83.3% vs. 70.8% and 45.8%, respectively). Moreover, the diagnostic predictive values were significantly higher for primary HPV with p16/Ki67 triage screening model (29.4% for PPV) compared to cytology with reflex HPV and cytology alone (21.3% and 22.9%, for PPV, respectively). The negative predictive value of primary HPV with p16/Ki67 triage for HPV-positive cases was very high (91.7% at CIN2+ threshold), which suggests the high safety of HPV-positive and p16/Ki67 negative women. The specificity for the detection of HSIL (CIN2+) of primary HPV triaging with p16/Ki67 was statistically significantly higher compared to cytology alone (47.8% vs. 35.1%) but was lower than for cytological screening with reflex HPV testing (61.9%). Our results also indicate a higher level of true screening test results in primary HPV with p16/Ki67 triage compared to cytology with reflex HPV and cytology alone screening models, due to the highest PLR (1.60 vs. 1.09 and 1.20, respectively) and lowest NLR (0.35 vs. 0.83 and 0.88) in this screening strategy.

Cytology is well known and easily available triaging option for HPV-positive women; however, it is suboptimal due to the lower sensitivity. This requires the use of a secondary screening tool that is more focused on the detection of cervical cancer precursors in HPV-positive patients. This approach was represented in our study by screening model no. 1, in which a highly specific and highly sensitive p16/Ki67 biomarker was incorporated as a triage test in women who had undergone primary HPV screening and had positive HPV status. 

Our study results are essentially consistent with the data from other reports on the evaluation of the diagnostic performance of p16/Ki67 dual-stained cytology for triaging HPV-positive cases [15,20,21,24]. All comparator studies involved groups of women <30 years, and most of them included women aged 21–24, as was designed in our investigation. 

Similarities with several studies in the sensitivity levels for detection HSIL (CIN2+) obtained in our screening model 1 were noted, including the study of the New Technologies for Cervical Cancer Screening group [24] (81.8%), the report of the Wentzensen et al. [20] (86.8%) in a comparable to our size study and the ATHENA sub-study trial—similarities with our results were observed only in the group with p16/Ki67 triaging N16/N18 HPV-positive test results (82.8%) [15]. A difference was found with the ATHENA study in the specificity for HSIL (CIN2+) detection (75.6% reported in the ATHENA). High consistency with our findings of diagnostic value levels for primary HPV with p16/Ki67 triage screening model was noted at the CIN2+ threshold with the data presented at the summary of safety and effectiveness data available at the FDA approval [21] (44.9% for specificity in HPV 16-positive cases; 81.8% for the sensitivity in N16/N18 HPV-positive cases; 93.8% for NPV). Positive predictive value levels for HSIL (CIN2+) detection fluctuate significantly in FDA registration document depending on the type of HPV-positivity sub-groups detected—HPV N16/N18, HPV 16, HPV 18 (19.2–44.0% vs. ours 27.0%). HPV test used in our study genotypes HPV16/18 and phenotypes 12 other HRHPV; however, we did not differentiate the results for HPV N16/N18 and HPV16/18 subgroups. Thus, it can be said that our results are averaged in this respect. Independently, in all the above-mentioned studies and in ours, high sensitivity was identified for primary HPV testing with p16/Ki67 triage in an HPV-positive group of women in detection HSIL (CIN2+).

High or very high levels of negative predictive value for detecting cervical cancer precursors, regardless of the threshold taken (HSIL(CIN2+) or HSIL(CIN3+)), in most other studies and in our observed at a level higher than 90% or higher than 95% [15,21,25], suggest high safety of women with HPV-positive status and with negative p16/Ki67 test result. This was strongly confirmed in the studies by Clarke et al. [26] on the 5-year cumulative risk of cervical precancer following p16/Ki67 triage of HPV-positive women, where that triaging approach provided better long-term risk stratification than cytology.

As part of our retrospective assessment of diagnostic performance in analyzed screening models was the determination of the HSIL (CIN2+) detection versus colposcopies need index. In screening model 3 with primary cytology alone, the lowest absolute number of colposcopies needed to perform for HSIL (CIN2+) detection was identified. However, the histologic HSIL detection index (CIN2+) per colposcopy rate in this model was higher than in the screening model based on primary HPV testing with p16/Ki67 triaging and based on cytology with reflex HPV. The best rate in the number of colposcopies needed to perform for detection of histologic HSIL was observed in screening model 1 with biomarker triage used. In both analyzed cytology-based screening models, 7 (in M2) and 13 (in M3) cases of HSIL (CIN2+) HRHPV14-positive and p16/Ki67-positive would not be detected.

We believe that promoting an effective cervical cancer screening with primary HPV in younger age groups of women, including women below 25, may be associated with building appropriate habits, which may result in a positive change in recently recorded increases in cancers rate among women 25–29 years. This may be particularly important in countries with very high HDI and with still too low HPV vaccination coverage and may be a factor making it independent of changes in sexual behavior [3]. Furthermore, primary HPV screening in young women might be a part of the catch-up vaccination strategy [27]. In cervical cancer systems with decentralized laboratory diagnostics, a significant reduction in the number of cytologic tests performed in HPV-based screening strategy, as well as potential replacing cytologic triage by p16/Ki67 dual-staining in a required pathologist evaluation, may result in the lower quality of cytological interpretation due to the decreasing number of experienced cytotechnicians. Hence, using the cytology screening model alone in a group of women <30 and/or <25 years may no longer be justified due to its insensitivity. When using M3 (cytology alone) in our analysis, all cases of HSIL (CIN2+) in women aged <25 years with preceding ASC-US and LSIL in cytology would not be detected in the first screening round (cytology follow-up in 1 year is recommended for these cases), which represented 12.5% of all HSIL (CIN2+) cases detected in women <30.

Strengths of this study: (1) One of the few studies evaluating the diagnostic value of p16/Ki67 triage in HPV-positive women <30 years who underwent primary HPV screening, including women below 25 years. (2) A thorough cervical disease ascertainment due to the extended colposcopic protocol used. (3) Our study gives an insight into screening tests results obtained in a private-based opportunistic cervical cancer screening. (4) All diagnostic tests were performed in the same specimen. (5) Immediate histologic correlation results due to the interval between colposcopy and abnormal screening test results not exceeding 3 months. (6) All p16/Ki67 tests were evaluated by a qualified gynecologic cytopathologist. Limitations of the study: (1) This study shows a post-hoc analysis. (2) We currently do not have a large enough group of patients to evaluate a diagnostic value at the CIN3+ threshold in the presented screening approaches. (3) Not all patients with indications for colposcopy decided to do it at the center. Due to different colposcopic protocols used, different histologic terminology and/or lack of p16 stain in cervical histologic specimens, the results of colposcopic biopsies performed outside the center were not included in the study.

## 5. Conclusions

In conclusion, our study showed that in women under 30 years, cervical cancer screening model based on primary HPV testing with p16/Ki67 dual-staining triage of HPV-positive women can be an effective screening approach in detecting cervical precancers and provide superior diagnostic value when compared with primary cytology with reflex HPV or primary cytology alone models. This investigation also demonstrated that young women below 25 years screened by primary HPV and triage with p16/Ki67 histologic HSIL biomarker might benefit from introducing this more sensitive screening algorithm, especially in the light of new data on the cervical cancer rates elevation in younger groups of women in countries with very high HDI.

## Figures and Tables

**Table 1:** Age-stratified LBC reporting rates (<30 and ≥30 years)—the baseline study group.

LBC Result	<30 y, No (%) ^1^	≥30 y, No (%) ^1^
NILM	3514 (91.3)	15,824 (94.7)
ASC-US	150 (3.9)	540 (3.2)
LSIL	162 (4.2)	258 (1.5)
ASC-H+	23 (0.60)	95 (0.57)
Total	3849 (100.0)	16,717 (100.0)

Abbreviations: LBC, liquid-based cytology; y, years; NILM, negative for intraepithelial lesion or malignancy; ASC-US, atypical squamous cells of undetermined significance; LSIL, low-grade squamous intraepithelial lesion; ASC-H+, cytology results: ASC-H (atypical squamous cells—cannot exclude HSIL), HSIL (high-grade squamous intraepithelial lesion) or AGC (atypical glandular cells); ^1^ % of all LBC results in the age group.

**Table 2:** Age-stratified LBC reporting rates (<30 and ≥30 years) in cases with HRHPV14, LBC, p16/Ki67 and histology results—the final study group selection.

LBC Result	HSIL (CIN2+) Result <30 y, No (%) ^1^	HSIL (CIN2+) Result ≥30 y, No (%) ^1^
NILM	4 (16.7)	13 (20.6)
ASC-US	8 (33.3)	15 (23.8)
LSIL	5 (20.8)	15 (23.8)
ASC-H+	7 (29.2)	20 (31.8)
Total	24 (100.0)	63 (100.0)

Abbreviations: LBC, liquid-based cytology; HRHPV14, 14 high-risk types human papillomavirus test; p16/Ki67, p16/Ki67 dual staining test; y, years; NILM, negative for intraepithelial lesion or malignancy; ASC-US, atypical squamous cells of undetermined significance; LSIL, low-grade squamous intraepithelial lesion; ASC-H+, cytology results: ASC-H (atypical squamous cells—cannot exclude HSIL), HSIL (high-grade squamous intraepithelial lesion) or AGC (atypical glandular cells); HSIL (CIN2+), histologic high-grade squamous intraepithelial lesion with quantification of cervical intraepithelial neoplasia in grade 2 or worse; ^1^ % of all HSIL (CIN2+) results in the age group.

**Table 3:** Biopsy results with HRHPV14 and p16/Ki67 status from baseline to final study group.

	No (%)	No (%)	Histology Result, No (%) ^3^			
HRHPV14 and p16/Ki67 Status	Results	Total Biopsy Results	Negative	LSIL (CIN1)	HSIL (CIN2)	HSIL (CIN3+)
HPV + ve	398 (30.1) ^1^	124 (96.1) ^2^	44 (35.5)	54 (43.6)	12 (9.7)	14 (11.3)
p16/Ki67	277 (69.6)	116 (93.6)	39 (33.6)	53 (45.7)	12 (10.3)	12 (10.3)
p16/Ki67 + ve	97 (35.0)	68 (58.6)	19 (27.9)	29 (42.7)	10 (14.7)	10 (14.7)
p16/Ki67 − ve	180 (65.0)	48 (41.4)	20 (41.7)	24 (50.0)	2 (4.2)	2 (4.2)
HPV − ve	923 (69.9) ^1^	5 (3.9) ^2^	2 (40.0)	3 (60.0)	0 (0.0)	0 (0.0)
p16/Ki67	39 (4.2)	5 (100.0)	2 (40.0)	3 (60.0)	0 (0.0)	0 (0.0)
p16/Ki67 + ve	7 (18.0)	4 (80.0)	1 (25.0)	3 (75.0)	0 (0.0)	0 (0.0)
p16/Ki67 − ve	32 (82.1)	1 (20.0)	1 (100.0)	0 (0.0)	0 (0.0)	0 (0.0)
Total	1321 (100.0) ^1^	129 (100.0) ^2^	46 (35.7)	57 (44.2)	12 (9.3)	14 (10.9)
p16/Ki67	316 (23.9)	121 (93.8)	41 (33.9)	56 (46.3)	12 (9.9)	12 (9.9)
p16/Ki67 + ve	104 (32.9)	72 (59.5)	20 (27.8)	32 (44.4)	10 (13.9)	10 (13.9)
p16/Ki67 − ve	212 (67.1)	49 (40.5)	21 (42.9)	24 (49.0)	2 (4.1)	2 (4.1)

Abbreviations: HRHPV14, 14 high-risk types human papillomavirus test; p16/Ki67, p16/Ki67 dual staining test; HPV, HRHPV14; +ve, positive; −ve, negative; LSIL (CIN1), histologic low-grade squamous intraepithelial lesion; HSIL (CIN2), histologic high-grade squamous intraepithelial lesion with quantification of cervical intraepithelial neoplasia in grade 2; HSIL (CIN3+), histologic high-grade squamous intraepithelial lesion with quantification of cervical intraepithelial neoplasia in grade 3 or worse; ^1^ % of total results; ^2^ % of total TBR results; ^3^ % of total biopsy results for the HRHPV14 and p16/Ki67 status defined in the first column.

**Table 4:** Age-stratified biopsy results (<25, 25–29 and <30 years) with HRHPV14 and p16/Ki67 status from baseline to final study group.

	No (%)	No (%)	Histology Result, No (%) ^3^			
HRHPV14 and p16/Ki67 Status	Results	Total Biopsy Results	Negative	LSIL (CIN1)	HSIL (CIN2)	HSIL (CIN3+)
<25 y	413 (31.3) ^1^	29 (22.5) ^2^	8 (27.6)	15 (51.7)	5 (17.2)	1 (3.5)
HPV + ve	137 (33.2)	27 (93.1)	7 (25.9)	14 (51.9)	5 (18.5)	1 (3.7)
p16/Ki67	98 (71.5)	25 (92.6)	6 (24.0)	13 (52.0)	5 (20.0)	1 (4.0)
p16/Ki67 + ve	28 (28.6)	16 (64.0)	4 (25.0)	7 (43.8)	4 (25.0)	1 (6.3)
p16/Ki67 − ve	70 (71.4)	9 (36.0)	2 (22.2)	6 (37.5)	1 (11.1)	0 (0.0)
HPV − ve	276 (66.8)	2 (6.9)	1 (50.0)	1 (50.0)	0 (0.0)	0 (0.0)
p16/Ki67	11 (4.0)	2 (100.0)	1 (50.0)	1 (50.0)	0 (0.0)	0 (0.0)
p16/Ki67 + ve	2 (18.2)	2 (100.0)	1 (50.0)	1 (50.0)	0 (0.0)	0 (0.0)
p16/Ki67 − ve	9 (81.8)	0 (0.0)	0 (0.0)	0 (0.0)	0 (0.0)	0 (0.0)
25–29 y	908 (68.7) ^1^	100 (77.5) ^2^	38 (38.0)	42 (42.0)	7 (7.0)	13 (13.0)
HPV + ve	261 (28.8)	97 (97.0)	37 (38.1)	40 (41.2)	7 (7.2)	13 (13.4)
p16/Ki67	179 (68.6)	91 (93.8)	33 (36.3)	40 (44.0)	7 (7.7)	11 (12.1)
p16/Ki67 + ve	69 (38.6)	52 (57.1)	15 (28.9)	22 (42.3)	6 (11.5)	9 (17.3)
p16/Ki67 − ve	110 (61.5)	39 (42.9)	18 (46.2)	18 (46.2)	1 (2.6)	2 (5.1)
HPV − ve	647 (71.3)	3 (3.0)	1 (33.3)	2 (66.7)	0 (0.0)	0 (0.0)
p16/Ki67	28 (4.3)	3 (100.0)	1 (33.3)	2 (66.7)	0 (0.0)	0 (0.0)
p16/Ki67 + ve	5 (17.9)	2 (66.7)	0 (0.0)	2 (100.0)	0 (0.0)	0 (0.0)
p16/Ki67 − ve	23 (82.1)	1 (33.3)	1 (100.0)	0 (0.0)	0 (0.0)	0 (0.0)
Total < 30 y	1321 (100.0) ^1^	129 (100.0) ^2^	46 (35.7)	57 (44.2)	12 (9.3)	14 (10.9)
HPV + ve	398 (30.1)	124 (96.1)	44 (35.5)	54 (43.6)	12 (9.7)	14 (11.3)
p16/Ki67	277 (69.6)	116 (93.6)	39 (33.6)	53 (45.7)	12 (10.3)	12 (10.3)
p16/Ki67 + ve	97 (35.0)	68 (58.6)	19 (27.9)	29 (42.7)	10 (14.7)	10 (14.7)
p16/Ki67 − ve	180 (65.0)	48 (41.4)	20 (41.7)	24 (50.0)	2 (4.2)	2 (4.2)
HPV − ve	923 (69.9)	5 (3.9)	2 (40.0)	3 (60.0)	0 (0.0)	0 (0.0)
p16/Ki67	39 (4.2)	5 (100.0)	2 (40.0)	3 (60.0)	0 (0.0)	0 (0.0)
p16/Ki67 + ve	7 (18.0)	4 (80.0)	1 (25.0)	3 (75.0)	0 (0.0)	0 (0.0)
p16/Ki67 − ve	32 (82.1)	1 (20.0)	1 (100.0)	0 (0.0)	0 (0.0)	0 (0.0)

Abbreviations: HRHPV14, 14 high-risk types human papillomavirus test; p16/Ki67, p16/Ki67 dual staining test; y, years; HPV, HRHPV14; +ve, positive; −ve, negative; LSIL (CIN1), histologic low-grade squamous intraepithelial lesion; HSIL (CIN2), histologic high-grade squamous intraepithelial lesion with quantification of cervical intraepithelial neoplasia in grade 2; HSIL (CIN3+), histologic high-grade squamous intraepithelial lesion with quantification of cervical intraepithelial neoplasia in grade 3 or worse; ^1^ % of total results; ^2^ % of total biopsy results; ^3^ % of total biopsy results for the HRHPV14 and p16/Ki67 status defined in the first column.

**Table 5:** Biopsy results with preceding LBC diagnosis.

	No (%) ^1^	No (%) ^2^	Histology Result, No (%) ^3^			
LBC Result	Results	Total Biopsy Results	Negative	LSIL (CIN1)	HSIL (CIN2)	HSIL (CIN3+)
NILM	3514 (91.3)	19 (15.7)	11 (57.9)	4 (21.1)	4 (21.1)	0 (0.0)
ASC-US	150 (3.9)	46 (38.0)	18 (39.1)	20 (43.5)	3 (6.5)	5 (10.9)
LSIL	162 (4.2)	46 (38.0)	11 (23.9)	30 (65.2)	3 (6.5)	2 (4.4)
ASC-H+	23 (0.60)	10 (8.3)	1 (10.0)	2 (20.0)	2 (20.0)	5 (50.0)
Total	3849 (100.0)	121 (100.0)	41 (33.9)	56 (46.3)	12 (9.9)	12 (9.9)

Abbreviations: LBC, liquid-based cytology; NILM, negative for intraepithelial lesion or malignancy; ASC-US, atypical squamous cells of undetermined significance; LSIL, low-grade squamous intraepithelial lesion; ASC-H+, cytology results: ASC-H (atypical squamous cells—cannot exclude HSIL), HSIL (high-grade squamous intraepithelial lesion) or AGC (atypical glandular cells); LSIL (CIN1), histologic low-grade squamous intraepithelial lesion; HSIL (CIN2), histologic high-grade squamous intraepithelial lesion with quantification of cervical intraepithelial neoplasia in grade 2; HSIL (CIN3+), histologic high-grade squamous intraepithelial lesion with quantification of cervical intraepithelial neoplasia in grade 3 or worse; ^1^ % of all LBC results; ^2^ % of all total biopsy results; ^3^ % of total biopsy results for the LBC status defined in the first.

**Table 6:** Age-stratified biopsy results (<25, 25–29 and <30 years) with preceding LBC diagnosis.

	No (%)	No (%)	Histology Result, No (%) ^3^			
LBC Result	Results	Total Biopsy Results	Negative	LSIL (CIN1)	HSIL (CIN2)	HSIL (CIN3+)
<25 y	109 (34.5) ^1^	27 (22.3) ^2^	7 (25.9)	14 (51.9)	5 (18.5)	1 (3.7)
NILM	37 (34.0)	6 (22.2)	3 (50.0)	1 (16.7)	2 (33.3)	0 (0.0)
ASC-US	29 (26.6)	11 (40.7)	3 (27.3)	6 (54.6)	2 (18.2)	0 (0.0)
LSIL	40 (36.7)	8 (29.6)	1 (12.5)	6 (75.0)	1 (12.5)	0 (0.0)
ASC-H+	3 (2.8)	2 (7.4)	0 (0.0)	1 (50.0)	0 (0.0)	1 (50.0)
25–29 y	207 (65.5) ^1^	94 (77.7) ^2^	34 (36.2)	42 (44.7)	7 (7.5)	11 (11.7)
NILM	84 (40.6)	13 (13.8)	8 (61.5)	3 (23.1)	2 (15.4)	0 (0.0)
ASC-US	48 (23.2)	35 (37.2)	15 (42.9)	14 (40.0)	1 (2.9)	5 (14.3)
LSIL	62 (30.0)	38 (40.4)	10 (26.3)	24 (63.2)	2 (5.3)	2 (5.3)
ASC-H+	13 (6.3)	8 (8.5)	1 (12.5)	1 (12.5)	2 (25.0)	4 (50.0)
Total < 30 y	316 (100.0) ^1^	121 (100.0) ^2^	41 (33.9)	56 (46.3)	12 (9.9)	12 (9.9)
NILM	121 (38.3)	19 (15.7)	11 (57.9)	4 (21.1)	4 (21.1)	0 (0.0)
ASC-US	77 (24.4)	46 (38.0)	18 (39.1)	20 (43.5)	3 (6.5)	5 (10.9)
LSIL	102 (32.3)	46 (38.0)	11 (23.9)	30 (65.2)	3 (6.5)	2 (4.4)
ASC-H+	16 (5.1)	10 (8.3)	1 (10.0)	2 (20.0)	2 (20.0)	5 (50.0)

Abbreviations: LBC, liquid-based cytology; HRHPV14, 14 high-risk types human papillomavirus test; p16/Ki67, p16/Ki67 dual staining test; y, years; NILM, negative for intraepithelial lesion or malignancy; ASC-US, atypical squamous cells of undetermined significance; LSIL, low-grade squamous intraepithelial lesion; ASC-H+, cytology results: ASC-H (atypical squamous cells—cannot exclude HSIL), HSIL (high-grade squamous intraepithelial lesion) or AGC (atypical glandular cells); LSIL (CIN1), histologic low-grade squamous intraepithelial lesion; HSIL (CIN2), histologic high-grade squamous intraepithelial lesion with quantification of cervical intraepithelial neoplasia in grade 2; HSIL (CIN3+), histologic high-grade squamous intraepithelial lesion with quantification of cervical intraepithelial neoplasia in grade 3 or worse; ^1^ % of all LBC results; ^2^ % of all total biopsy results; ^3^ % of total biopsy results for the LBC status defined in the first column.

**Table 7:** Age-stratified (<25, 25–29 and <30 years) LBS results.

	No (% of Total Results in the Age Group)	HRHPV14 and p16/Ki67 Results in Cytology Groups, No (% of LBS Results for LBC Diagnosis Defined in the First Column)					
		HRHPV14 + ve			HRHPV14 − ve		
LBC Status in Age Groups	Cases	p16/Ki67 + ve	p16/Ki67 − ve	Total	p16/Ki67 + ve	p16/Ki67 − ve	Total
<25 y total	109 (100.0)						
NILM	37 (34.0)	11 (29.7)	24 (64.9)	35 (94.6)	0 (0.0)	2 (5.4)	2 (5.4)
ASC-US	29 (26.6)	7 (24.1)	16 (55.2)	23 (79.3)	1 (3.4)	5 (17.2)	6 (20.7)
LSIL	40 (36.7)	8 (20.0)	29 (72.5)	37 (92.5)	1 (2.5)	2 (5.0)	3 (7.5)
ASC-H+	3 (2.8)	2 (66.7)	1 (33.3)	3 (100.0)	0 (0.0)	0 (0.0)	0 (0.0)
25–29 y total	207 (100.0)						
NILM	84 (40.6)	18 (21.4)	55 (65.5)	73 (86.9)	0 (0.0)	11 (13.1)	11 (13.1)
ASC-US	48 (23.2)	20 (41.7)	21 (43.8)	41 (85.4)	2 (4.2)	5 (10.4)	7 (14.6)
LSIL	62 (30.0)	21 (33.9)	32 (51.6)	53 (85.5)	2 (3.2)	7 (11.3)	9 (14.5)
ASC-H+	13 (6.3)	10 (76.9)	2 (15.4)	12 (92.3)	1 (7.7)	0 (0.0)	1 (7.7)
<30 y total	316 (100.0)						
NILM	121 (38.3)	29 (24.0)	79 (65.3)	108 (89.3)	0 (0.0)	13 (10.7)	13 (10.7)
ASC-US	77 (24.4)	27 (35.1)	37 (48.1)	64 (83.1)	3 (3.9)	10 (13.0)	13 (16.9)
LSIL	102 (32.3)	29 (28.4)	61 (59.8)	90 (88.2)	3 (2.9)	9 (8.8)	12 (11.8)
ASC-H+	16 (5.1)	12 (75.0)	3 (18.8)	15 (93.8)	1 (6.3)	0 (0.0)	1 (6.3)

Abbreviations: LBS, liquid-based screening; HRHPV14, 14 high-risk types human papillomavirus test; p16/Ki67, p16/Ki67 dual staining test; LBC, liquid-based cytology; +ve, positive; −ve, negative; y, years; NILM, negative for intraepithelial lesion or malignancy; ASC-US, atypical squamous cells of undetermined significance; LSIL, low-grade squamous intraepithelial lesion; ASC-H+, cytology results: ASC-H (atypical squamous cells—cannot exclude HSIL), HSIL (high-grade squamous intraepithelial lesion) or AGC (atypical glandular cells).

**Table 8:** Age-stratified (<25, 25–29 and <30 years) number of biopsy results in cytology groups.

	No (% of Total Results in <30 y Group)		Cytology Result, No (% of Total Biopsy Result)			
Age Group, Years	Cytology Results	Biopsy Results	NILM	ASC-US	LSIL	ASC-H+
<25	109 (34.5)	27 (22.3)	6 (22.2)	11 (40.7)	8 (29.6)	2 (7.4)
25–29	207 (65.5)	94 (77.7)	13 (13.8)	35 (37.2)	38 (40.4)	8 (8.5)
<30 (total)	316 (100.0)	121 (100.0)	19 (15.7)	46 (38.0)	46 (38.0)	10 (8.3)

Abbreviations: y, years; NILM, negative for intraepithelial lesion or malignancy; ASC-US, atypical squamous cells of undetermined significance; LSIL, low-grade squamous intraepithelial lesion; ASC-H+, cytology results: ASC-H (atypical squamous cells—cannot exclude HSIL), HSIL (high-grade squamous intraepithelial lesion) or AGC (atypical glandular cells).

**Table 9:** Age-stratified (<25, 25–29 and <30 years) LBS results for cases with biopsy results.

	No (% of Total Results in the Age Group)	HRHPV14 and p16/Ki67 Results in Cytology Groups, No (% of LBS Results for LBC Diagnosis Defined in the First Column)					
		HRHPV14 + ve			HRHPV14 − ve		
LBC Status in Age Groups	Total Biopsy Results	p16/Ki67 + ve	p16/Ki67 − ve	Total	p16/Ki67 + ve	p16/Ki67 − ve	Total
<25 y total	27 (100.0)						
NILM	6 (22.2)	6 (100.0)	0 (0.0)	6 (100.0)	0 (0.0)	0 (0.0)	0 (0.0)
ASC-US	11 (40.7)	6 (54.6)	4 (36.4)	10 (90.9)	1 (9.1)	0 (0.0)	1 (9.1)
LSIL	8 (29.6)	3 (37.5)	4 (50.0)	7 (87.5)	1 (12.5)	0 (0.0)	1 (12.5)
ASC-H+	2 (7.4)	1 (50.0)	1 (50.0)	2 (100.0)	0 (0.0)	0 (0.0)	0 (0.0)
25–29 y total	94 (100.0)						
NILM	13 (13.8)	11 (84.6)	2 (15.4)	13 (100.0)	0 (0.0)	0 (0.0)	0 (0.0)
ASC-US	35 (37.2)	17 (48.6)	17 (48.6)	34 (97.1)	1 (2.9)	0 (0.0)	1 (2.9)
LSIL	38 (40.4)	19 (50.0)	17 (44.7)	36 (94.7)	1 (2.6)	1 (2.6)	2 (5.3)
ASC-H+	8 (8.5)	5 (62.5)	3 (37.5)	8 (100.0)	0 (0.0)	0 (0.0)	0 (0.0)
<30 y total	121 (100.0)						
NILM	19 (15.7)	17 (89.5)	2 (10.5)	19 (100.0)	0 (0.0)	0 (0.0)	0 (0.0)
ASC-US	46 (38.0)	23 (50.0)	21 (45.7)	44 (95.7)	2 (4.4)	0 (0.0)	2 (4.4)
LSIL	46 (38.0)	22 (47.8)	21 (45.7)	43 (93.5)	2 (4.4)	1 (2.2)	3 (6.5)
ASC-H+	10 (8.3)	6 (60.0)	4 (40.0)	10 (100.0)	0 (0.0)	0 (0.0)	0 (0.0)

Abbreviations: LBS, liquid-based screening; HRHPV14, 14 high-risk types human papillomavirus test; p16/Ki67, p16/Ki67 dual staining test; LBC, liquid-based cytology; +ve, positive; −ve, negative; y, years; NILM, negative for intraepithelial lesion or malignancy; ASC-US, atypical squamous cells of undetermined significance; LSIL, low-grade squamous intraepithelial lesion; ASC-H+, cytology results: ASC-H (atypical squamous cells—cannot exclude HSIL), HSIL (high-grade squamous intraepithelial lesion) or AGC (atypical glandular cells).

**Table 10:** Age-stratified biopsy results (<25, 25–29 and <30 years) biopsy results with preceding LBS results.

LBS Status in Age Groups	Histology Results, No (% of Total Biopsy Results for the LBC, HRHPV14 and p16/Ki67 Results Defined in the First Column)				
LBC with HRHPV14 and p16/Ki67 Status	Total	Negative	LSIL (CIN1)	HSIL (CIN2)	HSIL (CIN3+)
<25 years total	27 (22.3)	7 (25.9)	14 (51.9)	5 (18.5)	1 (3.7)
NILM	6 (22.2) ^1^	3 (50.0)	1 (16.7)	2 (33.3)	0 (0.0)
HPV + ve	6 (100.0)	3 (50.0)	1 (16.7)	2 (33.3)	0 (0.0)
p16/Ki67 + ve	6 (100.0)	3 (50.0)	1 (16.7)	2 (33.3)	0 (0.0)
p16/Ki67 − ve	0 (0.0)	0 (0.0)	0 (0.0)	0 (0.0)	0 (0.0)
HPV − ve	0 (0.0)	0 (0.0)	0 (0.0)	0 (0.0)	0 (0.0)
p16/Ki67 + ve	0 (0.0)	0 (0.0)	0 (0.0)	0 (0.0)	0 (0.0)
p16/Ki67 − ve	0 (0.0)	0 (0.0)	0 (0.0)	0 (0.0)	0 (0.0)
ASC-US	11 (40.7) ^1^	3 (27.3)	6 (54.6)	2 (18.2)	0 (0.0)
HPV + ve	10 (90.9)	2 (20.0)	6 (60.0)	2 (20.0)	0 (0.0)
p16/Ki67 + ve	6 (60.0)	1 (16.7)	3 (50.0)	2 (33.3)	0 (0.0)
p16/Ki67 − ve	4 (40.0)	1 (25.0)	3 (75.0)	0 (0.0)	0 (0.0)
HPV − ve	1 (9.1)	1 (100.0)	0 (0.0)	0 (0.0)	0 (0.0)
p16/Ki67 + ve	1 (100.0)	1 (100.0)	0 (0.0)	0 (0.0)	0 (0.0)
p16/Ki67 − ve	0 (0.0)	0 (0.0)	0 (0.0)	0 (0.0)	0 (0.0)
LSIL	8 (29.6) ^1^	1 (12.5)	6 (75.0)	1 (12.5)	0 (0.0)
HPV + ve	7 (87.5)	1 (14.3)	5 (71.4)	1 (14.3)	0 (0.0)
p16/Ki67 + ve	3 (42.9)	0 (0.0)	3 (100.0)	0 (0.0)	0 (0.0)
p16/Ki67 − ve	4 (57.1)	1 (25.0)	2 (50.0)	1 (25.0)	0 (0.0)
HPV − ve	1 (12.5)	0 (0.0)	1 (100.0)	0 (0.0)	0 (0.0)
p16/Ki67 + ve	1 (100.0)	0 (0.0)	1 (100.0)	0 (0.0)	0 (0.0)
p16/Ki67 − ve	0 (0.0)	0 (00)	0 (0.0)	0 (0.0)	0 (0.0)
ASC-H+	2 (7.4) ^1^	0 (0.0)	1 (50.0)	0 (0.0)	1 (50.0)
HPV + ve	2 (100.0)	0 (0.0)	1 (50.0)	0 (0.0)	1 (50.0)
p16/Ki67 + ve	1 (50.0)	0 (0.0)	0 (0.0)	0 (0.0)	1 (100.0)
p16/Ki67 − ve	1 (50.0)	0 (0.0)	1 (100.0)	0 (0.0)	0 (0.0)
HPV − ve	0 (0.0)	0 (0.0)	0 (0.0)	0 (0.0)	0 (0.0)
p16/Ki67 + ve	0 (0.0)	0 (0.0)	0 (0.0)	0 (0.0)	0 (0.0)
p16/Ki67 − ve	0 (0.0)	0 (0.0)	0 (0.0)	0 (0.0)	0 (0.0)
25–29 years total	94 (77.7)	34 (36.2)	42 (44.7)	7 (7.5)	11 (11.7)
NILM	13 (13.8) ^1^	8 (61.5)	3 (23.1)	2 (15.4)	0 (0.0)
HPV + ve	13 (100.0)	8 (61.5)	3 (23.1)	2 (15.4)	0 (0.0)
p16/Ki67 + ve	11 (84.6)	7 (63.6)	2 (18.2)	2 (18.2)	0 (0.0)
p16/Ki67 − ve	2 (15.4)	1 (50.0)	1 (50.0)	0 (0.0)	0 (0.0)
HPV − ve	0 (0.0)	0 (0.0)	0 (0.0)	0 (0.0)	0 (0.0)
p16/Ki67 + ve	0 (0.0)	0 (0.0)	0 (0.0)	0 (0.0)	0 (0.0)
p16/Ki67 − ve	0 (0.0)	0 (0.0)	0 (0.0)	0 (0.0)	0 (0.0)
ASC-US	35 (37.2) ^1^	15 (42.9)	14 (40.0)	1 (2.9)	5 (14.3)
HPV +ve	34 (97.1)	15 (44.1)	13 (38.2)	1 (2.9)	5 (14.7)
p16/Ki67 + ve	17 (50.0)	3 (17.7)	8 (47.1)	1 (5.9)	5 (29.4)
p16/Ki67 − ve	17 (50.0)	12 (70.6)	5 (29.4)	0 (0.0)	0 (0.0)
HPV − ve	1 (2.9)	0 (0.0)	1 (100.0)	0 (0.0)	0 (0.0)
p16/Ki67 + ve	1 (100.0)	0 (0.0)	1 (100.0)	0 (0.0)	0 (0.0)
p16/Ki67 − ve	0 (0.0)	0 (0.0)	0 (0.0)	0 (0.0)	0 (0.0)
LSIL	38 (40.4) ^1^	10 (26.3)	24 (63.2)	2 (5.3)	2 (5.3)
HPV + ve	36 (94.7)	9 (25.0)	23 (63.9)	2 (5.6)	2 (5.6)
p16/Ki67 + ve	19 (52.8)	4 (21.1)	11 (57.9)	2 (10.5)	2 (10.5)
p16/Ki67 − ve	17 (47.2)	5 (29.4)	12 (70.6)	0 (0.0)	0 (0.0)
HPV − ve	2 (5.3)	1 (50.0)	1 (50.0)	0 (0.0)	0 (0.0)
p16/Ki67 + ve	1 (50.0)	0 (0.0)	1 (100.0)	0 (0.0)	0 (0.0)
p16/Ki67 − ve	1 (50.0)	1 (100.0)	0 (0.0)	0 (0.0)	0 (0.0)
ASC-H+	8 (8.5) ^1^	1 (12.5)	1 (12.5)	2 (25.0)	4 (50.0)
HPV + ve	8 (100.0)	1 (12.5)	1 (12.5)	2 (25.0)	4 (50.0)
p16/Ki67 + ve	5 (62.5)	1 (20.0)	1 (20.0)	1 (20.0)	2 (40.0)
p16/Ki67 − ve	3 (37.5)	0 (0.0)	0 (0.0)	1 (33.3)	2 (66.7)
HPV − ve	0 (0.0)	0 (0.0)	0 (0.0)	0 (0.0)	0 (0.0)
p16/Ki67 + ve	0 (0.0)	0 (0.0)	0 (0.0)	0 (0.0)	0 (0.0)
p16/Ki67 − ve	0 (0.0)	0 (0.0)	0 (0.0)	0 (0.0)	0 (0.0)
Total < 30 years	121 (100.0)	41 (33.9)	56 (46.3)	12 (9.9)	12 (9.9)
NILM	19 (15.7) ^1^	11 (57.9)	4 (21.1)	4 (21.1)	0 (0.0)
HPV + ve	19 (100.0)	11 (57.9)	4 (21.1)	4 (21.1)	0 (0.0)
p16/Ki67 + ve	17 (89.5)	10 (58.8)	3 (17.7)	4 (23.5)	0 (0.0)
p16/Ki67 − ve	2 (10.5)	1 (50.0)	1 (50.0)	0 (0.0)	0 (0.0)
HPV − ve	0 (0.0)	0 (0.0)	0 (0.0)	0 (0.0)	0 (0.0)
p16/Ki67 + ve	0 (0.0)	0 (0.0)	0 (0.0)	0 (0.0)	0 (0.0)
p16/Ki67 − ve	0 (0.0)	0 (0.0)	0 (0.0)	0 (0.0)	0 (0.0)
ASC-US	46 (38.0) ^1^	18 (39.1)	20 (43.5)	3 (6.5)	5 (10.9)
HPV + ve	44 (95.7)	17 (38.6)	19 (43.2)	3 (6.8)	5 (11.4)
p16/Ki67 + ve	23 (52.3)	4 (17.4)	11 (47.8)	3 (13.0)	5 (21.7)
p16/Ki67 − ve	21 (47.7)	13 (61.9)	8 (38.1)	0 (0.0)	0 (00)
HPV − ve	2 (4.4)	1 (50.0)	1 (50.0)	0 (0.0)	0 (0.0)
p16/Ki67 + ve	2 (100.0)	1 (50.0)	1 (50.0)	0 (0.0)	0 (0.0)
p16/Ki67 − ve	0 (0.0)	0 (0.0)	0 (0.0)	0 (0.0)	0 (0.0)
LSIL	46 (38.0) ^1^	11 (23.9)	30 (65.2)	3 (6.5)	2 (4.4)
HPV + ve	43 (93.5)	10 (23.3)	28 (65.1)	3 (7.0)	2 (4.7)
p16/Ki67 + ve	22 (51.2)	4 (18.2)	14 (63.6)	2 (9.1)	2 (9.1)
p16/Ki67 − ve	21 (48.8)	6 (28.6)	14 (66.7)	1 (4.8)	0 (0.0)
HPV − ve	3 (6.5)	1 (33.3)	2 (66.7)	0 (0.0)	0 (0.0)
p16/Ki67 + ve	2 (66.7)	0 (0.0)	2 (100.0)	0 (0.0)	0 (0.0)
p16/Ki67 − ve	1 (33.3)	1 (100.0)	0 (0.0)	0 (0.0)	0 (0.0)
ASC-H+	10 (8.3) ^1^	1 (10.0)	2 (20.0)	2 (20.0)	5 (50.0)
HPV + ve	10 (100.0)	1 (10.0)	2 (20.0)	2 (20.0)	5 (50.0)
p16/Ki67 + ve	6 (60.0)	1 (16.7)	1 (16.7)	1 (16.7)	3 (50.0)
p16/Ki67 − ve	4 (40.0)	0 (0.0)	1 (25.0)	1 (25.0)	2 (50.0)
HPV − ve	0 (0.0)	0 (0.0)	0 (0.0)	0 (0.0)	0 (0.0)
p16/Ki67 + ve	0 (0.0)	0 (0.0)	0 (0.0)	0 (0.0)	0 (0.0)
p16/Ki67 − ve	0 (0.0)	0 (0.0)	0 (0.0)	0 (0.0)	0 (0.0)

Abbreviations: LBS, liquid-based screening; LBC, liquid-based cytology; HRHPV14, 14 high-risk types human papillomavirus test; p16/Ki67, p16/Ki67 dual staining test; HPV, HRHPV14; NILM, negative for intraepithelial lesion or malignancy; ASC-US, atypical squamous cells of undetermined significance; LSIL, low-grade squamous intraepithelial lesion; ASC-H+, cytology results: ASC-H (atypical squamous cells—cannot exclude HSIL), HSIL (high-grade squamous intraepithelial lesion) or AGC (atypical glandular cells); +ve, positive; −ve, negative; LSIL (CIN1), histologic low-grade squamous intraepithelial lesion; HSIL (CIN2), histologic high-grade squamous intraepithelial lesion with quantification of cervical intraepithelial neoplasia in grade 2; HSIL (CIN3+), histologic high-grade squamous intraepithelial lesion with quantification of cervical intraepithelial neoplasia in grade 3 or worse; ^1^ % of total biopsy results in the age group.

**Table 11:** Performance characteristics of analyzed screening models for HSIL (CIN2+) detection.

Parameter	Primary HPV with Reflex p16/Ki67 (M1)	Primary Cytology with Reflex HPV (M2)	Primary Cytology without Reflex Test (M3)
Sensitivity, %	83.3 (62.6–95.3)	70.8 (48.9–87.4)	45.8 (25.6–67.2)
Specificity, %	47.8 (37.3–58.5)	35.1 (25.6–45.4)	61.9 (51.4–71.5)
PPV, %	29.4 (19.0–41.7)	21.3 (12.9–31.8)	22.9 (12.0–37.3)
NPV, %	91.7 (80.0–97.7)	82.9 (67.9–92.9)	82.2 (71.5–90.2)
PLR	1.60 (1.23–2.08)	1.09 (81.2–1.47)	1.20 (0.73–1.99)
NLR	0.35 (0.14–0.87)	0.83 (0.42–1.64)	0.88 (0.59–1.31)

Abbreviations: HPV, 14 high-risk types human papillomavirus test; M1, model 1; M2, model 2; M3, model 3; PPV, positive predictive value; NPV, negative predictive value; PLR, positive likelihood ratio; NLR, negative likelihood ratio.

**Table 12:** Performance characteristics of analyzed screening models: loss/detection of HSIL (CIN2+) vs. number of colposcopies needed.

Screening Model	Number of HSIL (CIN2+) Lost/Detected	Number of Colposcopies Needed in Each Model	Number of Colposcopies Per HSIL (CIN2+) Detection
Primary HPV with reflex p16/Ki67 (M1)	4/20	68	3,4
Primary cytology with reflex HPV (M2)	7/17	80	4,7
Primary cytology without reflex test (M3)	13/11	48	4,4

Abbreviations: HSIL (CIN2+), histologic high-grade squamous intraepithelial lesion with quantification of cervical intraepithelial neoplasia in grade 2 or worse; HPV, 14 high-risk types human papillomavirus test; M1, model 1; M2, model 2; M3, model 3.

## Data Availability

The data presented in this study are available on request from the corresponding author.
